# Gene Therapies for Primary Immune Deficiencies

**DOI:** 10.3389/fimmu.2021.648951

**Published:** 2021-02-25

**Authors:** Lisa A. Kohn, Donald B. Kohn

**Affiliations:** ^1^Division of Pediatric Allergy and Immunology, Department of Pediatrics, David Geffen School of Medicine, University of California, Los Angeles, Los Angeles, CA, United States; ^2^Division of Pediatric Hematology/Oncology, Department of Pediatrics, David Geffen School of Medicine, University of California, Los Angeles, Los Angeles, CA, United States

**Keywords:** primary immune deficiency, gene therapy, retroviral vector, lentiviral vector, hematopoietic stem cell

## Abstract

Gene therapy is an innovative treatment for Primary Immune Deficiencies (PIDs) that uses autologous hematopoietic stem cell transplantation to deliver stem cells with added or edited versions of the missing or malfunctioning gene that causes the PID. Initial studies of gene therapy for PIDs in the 1990–2000's used integrating murine gamma-retroviral vectors. While these studies showed clinical efficacy in many cases, especially with the administration of marrow cytoreductive conditioning before cell re-infusion, these vectors caused genotoxicity and development of leukoproliferative disorders in several patients. More recent studies used lentiviral vectors in which the enhancer elements of the long terminal repeats self-inactivate during reverse transcription (“SIN” vectors). These SIN vectors have excellent safety profiles and have not been reported to cause any clinically significant genotoxicity. Gene therapy has successfully treated several PIDs including Adenosine Deaminase Severe Combined Immunodeficiency (SCID), X-linked SCID, Artemis SCID, Wiskott-Aldrich Syndrome, X-linked Chronic Granulomatous Disease and Leukocyte Adhesion Deficiency-I. In all, gene therapy for PIDs has progressed over the recent decades to be equal or better than allogeneic HSCT in terms of efficacy and safety. Further improvements in methods should lead to more consistent and reliable efficacy from gene therapy for a growing list of PIDs.

## Introduction

Allogeneic hematopoietic stem cell transplantation (HSCT) has been a definitive therapy for the most severe PIDs over the past 4–5 decades. Replacement of the patient's hematopoietic stem cells (HSC) that carry the defective PID-causing gene with allogeneic HSC from a healthy donor can lead to production of genetically normal blood cells and restore immunity. However, immunological differences between the patient and the HSC donor may lead to significant complications, including rejection of the donor HSC graft or attack by the transferred donor's immune cells against the recipient – graft vs. host disease (GVHD). In addition to pre-transplant myelosuppressive agents that open up space in the HSC niche, potent immune suppressive agents are often administered to minimize risks of donor cell rejection. Post-transplant, immune suppression is often given to prevent development of GVHD. While allogeneic HSCT is a mainstay of treatment and has had progressively improved outcomes, the immunological complications remain a major source of morbidity and potential mortality.

**Figure 1:**
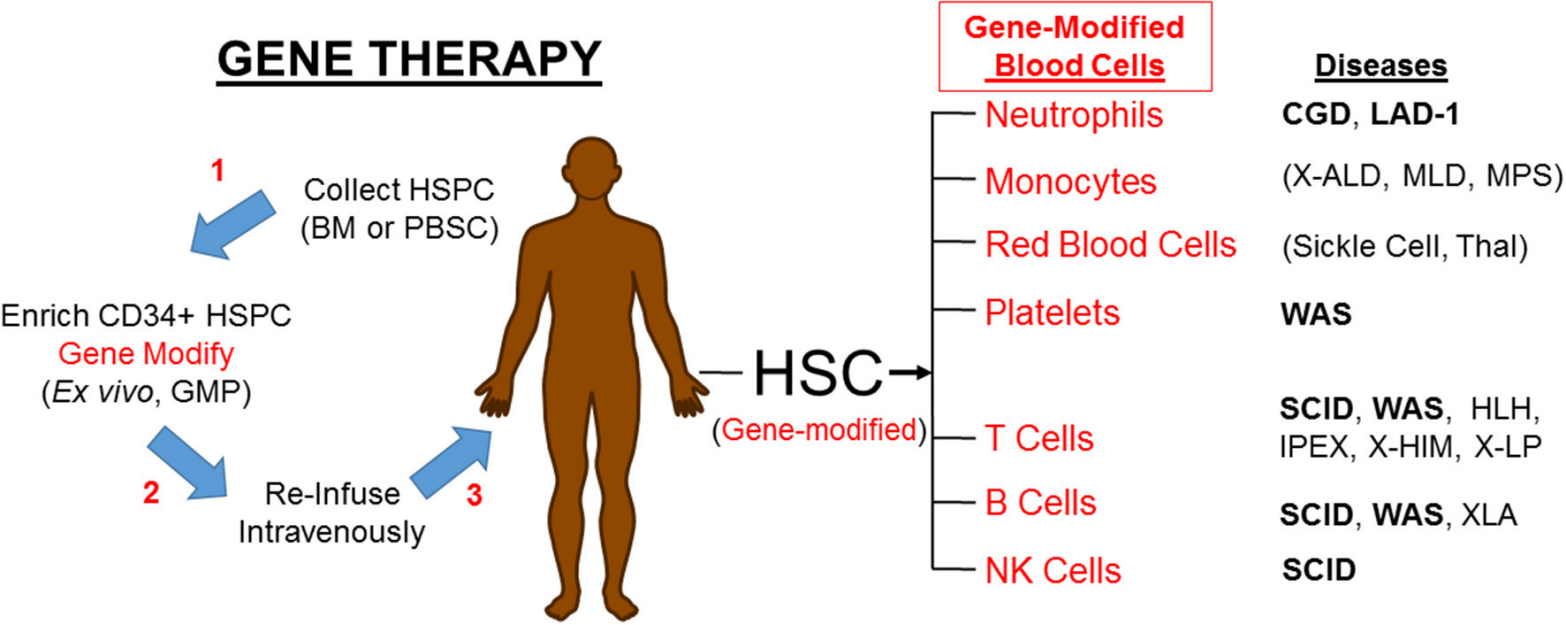
Gene therapy corrects PIDs by restoring the affected blood cells. HSPC, hematopoietic progenitor and stem cells; BM, bone marrow; PBSC, peripheral blood stem cells; GMP, good manufacturing practice laboratory; HSC, hematopoietic stem cells; CGD, Chronic Granulomatous Disease; LAD-1, Leukocyte Adhesion Deficiency-I; X-ALD, X-linked Adrenoleukodystrophy; MLD, metachromatic Leukodystrophy; MPS, Mucopolysaccharidoses; Thal, α- or β-Thalassemia; WAS, Wiskott-Aldrich Syndrome; SCID, Severe Combined Immune Deficiency; HLH, Hemophagocytic Lymphohistiocytosis; IPEX, Immune Deficiency Polyendocrinopathy, X-Linked; XHIM, X-linked Hyper IgM; XLP, X linked Lymphoproliferative. Diseases in bold font are currently in clinical trials. Parenthesis () mark non-PIDs.

If PIDs can be treated by engrafting allogeneic HSC, it should be possible to treat PIDs by engrafting autologous HSC that have been gene corrected, either by gene addition using a viral vector (as will be discussed here) or by gene editing (not discussed here) to regain normal function (Figure 1). Upon re-engraftment of the gene-corrected stem cells and production of the relevant mature immune effector cells with appropriate expression of the corrective gene, gene therapy should provide beneficial impact on the PID without the immunological complications of allogeneic HSCT. Recently improved genetic and molecular understanding of mechanisms of PIDs has led to identification of the underlying defective genes, thereby increasing the PIDs that are eligible to be gene therapy candidates.

While gene therapy is conceptually attractive, it took more than a decade for any evidence of efficacy against a PID to be realized in clinical trials (1, 2). Since that time, through iterative improvements, gene therapy is being successfully applied to treat a growing list of PIDs, as discussed here (Figure 1). Gene therapy is transitioning from experimental academic based trials to licensed drug products that are routinely beneficial [see (3) for a recent comprehensive review].

## Adenosine Deaminase Severe Combined Immune Deficiency

Absence of adenosine deaminase (ADA) enzyme in a SCID patient was first deduced to be a cause of human SCID in 1972 by Eloise Giblett (4). Subsequent studies determined that in the absence of ADA, high levels of its substrates, adenosine and deoxyadenosine, accumulate systemically, resulting in lymphotoxicity by disturbing nucleoside metabolism (5, 6). ADA SCID patients typically present with pan-lymphopenia (T-/B-/NK- SCID) with risks for opportunistic infections. Additionally, some ADA SCID patients may have extra-immune system complications, such as pulmonary alveolar proteinosis presenting in the neonatal period, hearing impairment, neurodevelopmental disorders, attention deficit disorders, as well as GI or skeletal abnormalities (7).

An early treatment for ADA SCID was frequent transfusions of red blood cells (RBC) that transferred ADA enzyme contained in the RBCs to catabolize adenosine nucleosides and allow survival of some lymphocytes (8). This led to the development of an ADA enzyme replacement therapy (ERT) composed of bovine ADA, purified biochemically then conjugated to polyethylene glycol (PEG-ADA) (9). Intramuscular injections of PEG-ADA led to better detoxification of adenine metabolites than with RBC transfusions, although immune reconstitution was often incomplete with sub-normal levels of lymphocytes restored (10). In recent years, a recombinant form of pegylated ADA enzyme has become available.

HSCT for ADA SCID was first reported by Parkman et al. (11), with three patients successfully treated with marrow infusion from HLA matched siblings with no preceding cytoreductive conditioning. Non-conditioned sibling transplants remain a treatment of choice when available. Results from transplants for ADA SCID with other donors have been less satisfactory, with the largest multi-center series reporting 66% survival with matched unrelated donors and only 43% with haplo-identical donors (12). This study was based on transplants done one to three decades ago; more recent outcomes are likely improved by early diagnosis by newborn screening, improved transplant conditioning and graft manipulation techniques, and better medical therapies for infections (13). Nonetheless, gene therapy has been pursued for ADA SCID to provide better survival and immune outcomes, especially for patients lacking matched sibling donors because of the expectation that even a few gene-corrected stem cells could produce sufficient lymphocytes to reconstitute immunity.

ADA SCID was the first human disease to be approached by gene therapy (excluding an early attempt to treat beta-thalassemia by injection of a plasmid directly into bone marrow). The first clinical gene therapy for ADA SCID was performed at the National Institutes of Health in 1990, targeting a normal ADA gene into peripheral blood lymphocytes (14). There was persistence of ADA gene-marked lymphocytes for more than a decade after the gene therapy, demonstrating the longevity of these peripheral cells, despite *ex vivo* manipulation (15). While this approach to gene therapy for ADA SCID using transduced peripheral blood lymphocytes has not been extensively used after this first study, it did presage the development of Chimeric Antigen Receptor-modified (CAR) T lymphocytes, which has revolutionized the treatment of B lineage malignancies.

Gene therapy for ADA SCID was first made efficacious through studies performed in Milan, Italy (2). A gamma-retroviral vector was used to transduce bone marrow CD34+ cells. Reduced intensity conditioning with busulfan was administered prior to reinfusion of the cells, dosed at 4 mg/kg, approximately 25% of the full dose used for complete cytoablation. Cytoreductive conditioning opened sufficient “space” in the marrow stem cell niche to facilitate engraftment of the gene-modified long-term stem cells and is well-tolerated. These gene-corrected HSC can engraft, proliferate and produce ADA gene containing lymphocytes leading to immune restoration. The first report described two patients who demonstrated clinically effective levels of immune reconstitution with stable persistence of the gene-corrected stem cells (2). These investigators in Italy and others in the U.K. and the U.S. reported that, in at least 40 ADA SCID patients, gene therapy led to immune reconstitution for the majority of patients, with no incidents of vector-related adverse effects (16–22).

A gene therapy for ADA SCID has been approved by the European Medicines Agency (EMA), Strimvelis, which was derived from the work done at the Milan center. Unfortunately, one recipient of this gene therapy developed lymphoproliferation several years after treatment,^1^ representing the first case of insertional oncogenesis leading to clinically significant leukoproliferation in ADA SCID patients over 20 years of the use of gamma-retroviral vectors.

A lentiviral vector has also been studied for gene therapy for ADA SCID in the U.S. and the U.K (23). Forty eight of 50 patients responded with restoration of immunity sufficient to obviate a rescue allogeneic HSCT or resumption of enzyme replacement. In the US study, 90% of the patients had discontinued immunoglobulin replacement by 2 years after gene therapy, while in the EU study, 100% of the patients had discontinued immunoglobulin replacement by 3 years after gene therapy. There were no adverse effects from the vector. The high success rate of autologous gene therapy for ADA SCID led to a consensus report recommending that gene therapy may be considered equivalent in potential efficacy to a matched sibling donor transplant, due to the complete absence of graft vs. host disease with gene therapy and the use of reduced intensity conditioning without post-transplant immune suppression (24).

While the gene therapies described above have led to high rates of correction of the life-threatening immune deficiency, the other complications in ADA SCID, such as hearing loss and neurodevelopmental deficits, may not be corrected by any of the therapeutic interventions that only correct hematopoietic cells. It is hoped that early identification of ADA SCID patients by newborn screening, the prompt implementation of ADA ERT, and the subsequent curative treatment by HSCT or gene therapy may reduce these complications, but this is not known at present.

## X-linked Severe Combined Immune Deficiency

X-SCID is an X-linked recessive disease that results from pathogenic variations in the *IL2RG* gene (also known as the common gamma chain). X-SCID is the most common form of SCID, accounting for approximately one-third of cases (25). The common gamma chain protein is a component of the receptor for cytokines IL2, IL4, IL7, IL9, IL15, and IL21. X-SCID patients have the inability to develop T cells and NK cells, and B cells are present but non-functional (T-/B+/NK- SCID).

Initial gene therapy trials for X-SCID enrolled patients without an HLA-identical sibling donor, 10 patients in France and 10 patients in the United Kingdom (1, 26–29). Patients' CD34+ HSCs were transduced with Moloney murine leukemia virus-based gamma retroviral vectors containing normal *IL2RG* cDNA. These vectors retained the strong viral enhancer elements present in the long-terminal repeats (LTR) to drive high level expression of *IL2RG* throughout the lymphoid lineages. The majority of patients (18/20) had appropriate T cell reconstitution after gene therapy. But six of the patients in the initial trials experienced serious adverse events, developing T cell acute lymphoblastic leukemia 2–14 years after undergoing gene therapy (30–32). Gamma-retroviral vectors preferentially integrate into the 5' end of actively transcribed genes in the targeted HSC. In 4 of the patients, the leukemic clones showed retroviral vector integrated near the LIM domain-only 2 (*LMO2*) proto-oncogene, driving its over-expression, thought to be the leukemic initiating event.

After these patients experienced insertional oncogenesis, modifications to the next generation of gamma retroviral vectors focused on decreasing the ability of elements within the vectors to *trans*-activate neighboring endogenous proto-oncogenes (33). These next generation self-inactivating (“SIN”) retroviral vectors were used without conditioning to treat patients at 5 centers in the United States and Europe. The LTR enhancers are removed from the SIN vectors through a self-inactivation process during reverse transcription, and are replaced with internal promoters that promote expression of the *IL2RG* gene but lack enhancer activity that could *trans*-activate cellular genes near sites of integration. Studies of vector insertion sites in peripheral blood cells of X-SCID gene therapy patients receiving SIN gamma-retroviral vectors demonstrated less clustering at proto-oncogenes than in the cells from patients who received the original vectors (34). To date, no SIN vector has produced insertional oncogenesis in an X-SCID patient, demonstrating their improved safety profiles.

The more recent trials have differed in several ways in order to increase immune system recovery while avoiding vector-related complications. The autologous CD34+ HSCs source has been expanded to include either bone marrow or mobilized peripheral blood. The vectors used for the recent trials are SIN-lentiviral vectors, to further decrease the risk of insertional oncogenesis. Busulfan is being given at doses to reach target AUC of 20–30,000 ng/ml^*^h. Cells are commonly cryopreserved after transduction, serving two purposes. First, cryopreservation allows time for patients to receive pharmacokinetic-based adjustment of split dose reduced intensity conditioning with busulfan prior to infusion of their modified stem cells, to increase HSC engraftment and improve sustained production of B and NK lymphoid cells. Second, cryopreservation of the cell product at the completion of gene transduction culture allows cell products to undergo full release testing and then be shipped and infused at a remote site, as has been done for multi-center trials of gene therapy for β-thalassemia (35), Leukocyte Adhesion Deficiency-I (36) and others.

A recent publication described 8 infants with X-SCID treated with lentiviral vector based gene therapy with production of gene-corrected B, T and NK cells, improvement of IgG levels, and no evidence of clonal insertional events at short term follow up (37). Another publication described a phase 1/2 clinical trial that used a SIN-lentiviral vector with busulfan conditioning in five older X-SCID patients (ages 7–23 years old) who had previously undergone haplo-identical HSCT but had minimal or unsustained immune reconstitution (38). As of publication, these patients had clinical and immunologic improvement at short term follow-up. Ongoing studies are treating further X-SCID patients with SIN-lentiviral vectors and no vector-related complications have been reported (39).

## Artemis-Deficient Severe Combined Immune Deficiency

Pathogenic variations in *DCLREIC* (encoding the Artemis protein) cause autosomal recessive T-B-NK+ SCID (Art-SCID). Artemis is an endonuclease required to repair double stranded DNA breaks using non-homologous end joining. Artemis is used in the processes of V(D)J recombination that create a diverse repertoire of genes encoding B cell immunoglobulin receptors (and antibodies) and T cell receptors. Art-SCID patients are radiosensitive, with increased adverse effects after radiation or alkylating agents, due to their inability to repair double-stranded breaks. Like other forms of SCID, Art-SCID patients can be treated with HSCT, with patients receiving HSCT from matched sibling donors surviving at a rate of 85% and from haplo-identical donors at a survival rate of 65% (40). Unfortunately, treatment with HSCT is challenging because Art-SCID patients experience more severe adverse effects due to conditioning than in other forms of SCID, including endocrinological and nutritional deficiencies leading to growth delay. However, insufficient conditioning allows endogenous NK cells to interfere with donor cell engraftment and may lead to poor B cell reconstitution. Therefore, by gene modifying the patient's endogenous cells, ART-SCID patients may be able to benefit from reduced conditioning without the concern of NK rejection of the donor cells.

The first clinical trial to treat Art-SCID is currently enrolling patients, with 5 newly diagnosed patients and 3 patients previously treated with allogeneic HSCT reported in a meeting abstract (41). Patients received low dose busulfan prior to receiving CD34+ cells transduced by a self-inactivating lentiviral vector expressing *DCLRE1C* driven by its endogenous promoter. At short term follow up, 3 of 3 infant patients had multi-lineage vector integration, normal mitogen lymphocyte proliferation, and had been discharged from the hospital. Two of the patients acquired and recovered from viral infections (rhinovirus, CMV and rotavirus). Thus far, the only reported side effect has been autoimmune hemolytic anemia of uncertain etiology occurring early after treatment in two infants and one previously treated patient; this problem resolved after T cell immunity recovered. New vectors and gene editing strategies for gene therapy of Art-SCID are also being assessed by other investigators.

## Wiskott-Aldrich Syndrome

Wiskott Aldrich Syndrome (WAS) is an X-linked pan-hematopoietic defect that classically presents with bleeding (due to small platelets), eczema, and infections, and frequently involves autoimmunity and lymphoid neoplasms. The Wiskott Aldrich Syndrome Protein (WASP) is expressed only in hematopoietic cells and is an important regulator of actin cytoskeleton. WASP dysfunction impairs normal leukocyte migration, phagocytosis, creation of immune synapse, and intracellular signaling. Abnormal WASP also creates abnormalities of megakaryopoiesis, with micro-platelets and thrombocytopenia. Autoimmunity (including hemolytic anemia, vasculitis, arthritis, nephropathy, and inflammatory bowel disease) results from insufficient Treg functionality, defective apoptosis, and increased auto-reactive B cells (42). WAS patients may have a wide variety of infections due to the impact of WASP on multiple blood cell lineages. The underlying genetic defects in *WAS* are found throughout the 12 exons of the gene, with defects in the C terminus associated with more severe disease symptoms (43). Pathogenic *WAS* missense variants that lead to reduced expression of functional protein or abnormal protein typically cause clinically milder disease than do nonsense or frameshift variations that lead to an absence of WASP protein (44).

Patients with severe WAS (especially those who do not express WASP) typically have a life expectancy of <15 years in the absence of HSCT due to hemorrhage, malignancy and severe infection (45). HSCT offers curative treatment for WAS. HSCT typically has excellent outcomes with HLA-identical donors, with 5 year survival at >90%. But there may be higher morbidity and mortality with non-HLA identical donors and for patients older than 5 years when they undergo the procedure (46). A more recent review of 129 WAS patients undergoing allogeneic HSCT was reported by the Primary Immune Deficiency Consortium (PIDTC) (47). They reported overall survival of 91% and found that donor type did not make a significant difference for survival.

WAS, as monogenic primary immunodeficiency affecting only hematopoietic cells, is an excellent candidate for gene therapy but faces the challenge of needing sufficient gene expression in myeloid, lymphoid, and megakaryocytic lineages in order to correct all aspects of the pathology. The first clinical trial of gene therapy enrolled 10 patients with severe WAS in Germany beginning in 2006 (48). After conditioning with reduced intensity busulfan (8 mg/kg), patients received CD34+ cells transduced with an LTR-intact gamma retroviral vector containing a normal WAS cDNA. Initially, 9 out of 10 enrolled patients demonstrated improved T cell proliferation, antibody titers and increased platelet counts and size. However, despite initial success with improvement in both immune functioning and bleeding, at least 7 out of 10 patients developed leukemia due to insertional oncogenesis at the *LMO* and *MECOM* loci (49) while more recent reports have stated that oncogenesis occurred in 9 of 10 (50).

Subsequently, three clinical trials in the United States and Europe have used CD34+ peripheral blood stem cells transduced with a self-inactivating lentiviral vector containing a *WAS* cDNA with expression controlled by the endogenous *WAS* promoter [reviewed in (51–57)]. The four centers each used slightly different conditioning regiments, with busulfan dosed for non-myeloablative or myeloablative targets, fludarabine used at 60 or 120 mg/m^2^ and two of the four trials including anti-CD20 monoclonal.

These trials have treated at least 34 patients, with 91.5% aggregate survival (51). Despite WASP levels below normal, patients had improvement of immune function with increased T cell proliferation, vaccine antibody responses allowing discontinuation of immunoglobulin supplementation in most patients, and decreased infections. Although patients had mild increases of platelet counts and decreased incidents of severe bleeding events, some patients needed to continue to receive platelet transfusions episodically and had continued minor bleeding events. Patients with vector copy numbers > 2 copies per cell had better platelet reconstitution (>50,000/μl) (56). Thus far, no leukemia or abnormal clonal proliferation has been reported in WAS patients receiving the lentiviral-based gene therapy. These benefits of gene therapy for WAS demonstrated in pediatric populations have been extended to an adult WAS patient with severe chronic disease complications. Stable engraftment of gene-marked multi-lineage cells was achieved with improvement in T cell numbers and function, and improvement of multiple inflammatory complications (57).

## X-linked Chronic Granulomatous Disease

Chronic Granulomatous Disease (CGD) was recognized as a distinct disorder in patients who had recurrent infections and cutaneous and visceral granulomas without detectable micro-organisms, but with apparently intact antibody production and delayed hypersensitivity (T cell-mediated) responses (58). CGD is now understood to result from defects in the NADPH-dependent oxidase complex in phagocytic leukocytes that is responsible for creating a burst of anti-microbial oxygen radicals (59). In the absence of phagocytic oxidase, neutrophils can engulf microorganisms but not eradicate them, leading to persistence of infections, commonly due to *Staphylococcus, Serratia*, and *Aspergillus*. There are five critical oxidase protein components and genetic deficiency of any one may give the CGD phenotype. Pathogenic variations in the Gp91phox protein, encoded on the X-chromosome, are the most common genetic cause for CGD. X-linked Chronic Granulomatous Disease (XCGD) accounts for approximately 65% of CGD. While use of prophylactic antibiotics and antifungals and aggressive treatment of infections that do occur can reduce morbidity in CGD, nevertheless patients offer suffer recurrent infections, inflammatory complications, hospitalizations and early mortality.

Allogeneic HSCT can be curative for all forms of CGD and the development of reduced intensity conditioning protocols has led to relatively high rates of survival and correction of the immune deficiency (60). A recent retrospective review of 712 CGD patients transplanted at multiple centers over a 15 year period confirmed these observations, with median survival at 3 years of 85.7% and event-free survival of 75.8% (61). The PIDTC have reported that the presence of inflammatory bowel disease (IBD) complicating CGD did not affect transplant outcomes and IBD resolved by 2 years post-transplant in all affected (62). Nevertheless, there remain risks of rejection and graft vs. host disease, and some patients do not have a suitable family or unrelated donor available.

Hence gene therapy for CGD has been under study for almost three decades. Early gene therapy efforts for CGD in several trials were performed in the 1990's using gamma-retroviral vectors, but no cytoreductive conditioning (63). In general, these did not lead to any significant engraftment of gene corrected stem cells and hence no clinical benefits.

With the introduction of cytoreductive conditioning to XCGD gene therapy trials, engraftment of gene-corrected stem cells was achieved in two trials, using busulfan 10 mg/kg and busulfan 6.4 mg/kg and fludarabine 120 mg/m^2^, respectively (64, 65). One trial used G-CSF mobilized CD34+ peripheral blood stem cells transduced with a gamma-retroviral vector with a potent LTR enhancer/promoter element (from the Spleen Focus Forming Virus) and busulfan conditioning at 8 mg/kg. They achieved clinically relevant levels of engraftment of gene-corrected stem cells and sufficient functioning neutrophils to lead to clearance of severe treatment resistant infections (66). However, all three of the subjects subsequently developed myeloproliferation as a result of insertional oncogenesis by the retroviral LTR enhancers (67, 68).

More recently, clinical trials of gene therapy for XCGD have been performed using a lentiviral vector. Based on the myeloproliferative complications that occurred using a gamma-retroviral vector, the lentiviral vector was designed to use enhancers and promoters from genes expressed during myeloid differentiation [c-fes (*FES*) and Cathepsin-G (*CTSG*)], to prevent trans-activation of proto-oncogenes at the stem and progenitor stages that are vulnerable to transformation, but express sufficient levels of the needed oxidase in mature myeloid cells (69). As recently reported, a majority of the patients treated by gene therapy for XCGD using the myeloid-specific lentiviral vector have shown stable engraftment of gene-corrected stem cells with persistence of vector-containing neutrophils and >10% DHR+ cells for 2–4 years (70). No vector-related complications were observed. The thresholds of gene-corrected neutrophils for normal control of infections and absence of inflammatory complications are not known. Studies of female XCGD carriers found that the sub-group with CGD-type infections had a median 8% DHR+ neutrophils whereas those with auto-inflammatory complications had a median 38% DHR+ neutrophils (71).

However, as mentioned in Kohn et al. (70), three subsequent pediatric XCGD patients suffered the loss of most of the gene marked cells in the first 3–6 months after gene therapy, in contrast to the stable persistence of therapeutic levels of gene marked cells of the adult patients that extended beyond 2 years after gene therapy. The basis for the loss of engrafted stem cells is unexplained. One possibility is that an immune response developed to the normal gp91phox gene product that may be seen as a neo-antigen by the immune system, leading to rejection of gene-containing cells. Alternatively, the hematopoietic stem cells and their bone marrow microenvironment niche of CGD patients are known to be under continual stress from CGD induced inflammation, potentially impairing stem cell numbers, stem cell survival during *ex vivo* culture, or their engraftment (72). It is important to distinguish between these or other possibilities to make gene therapy for XCGD more consistently reliable.

Jofra Hernandez et al. (73) recently reported the development of leukemia in a small percentage of XCGD mice receiving a lentiviral vector for gene therapy bone marrow transplant studies. They speculate that the inherent inflammatory milieu of the XCGD marrow microenvironment may contribute to oncogenicity. Fortunately, this complication has not been seen in human XGGD gene therapy subjects to date; it is not known if the specific lentiviral vector construct used by Hernandez and colleagues heightened this risk, but it indicates that patients will need monitoring for the emergence of leukoproliferative problems.

It may be concluded that there has been excellent progress with gene therapy for XCGD with multiple patients receiving clinical benefits, but several issues remaining to be resolved to improve the outcomes.

## Leukocyte Adhesion Deficiency-I

LAD-1 is an autosomal recessive disorder with defects in the gene encoding a critical leukocyte adhesion molecule (*ITGB2* encoding CD18) required for neutrophils to adhere to ICAM molecules on endothelium to leave the bloodstream and move to sites of infections (74, 75). LAD-1 patients may present in infancy due to delayed separation of the umbilical cord which may be complicated by the development of omphalitis. LAD-I patients have high frequencies of skin and mucosal infections, inflammatory skin lesions, and severe gingivitis, which have been shown to respond to treatment with Ustekinumab, a monoclonal antibody that blocks IL-12 and IL-23 (76). Patients with severe absence of CD18 expression (<2%) have a very high rate of early mortality, and patients with partial deficiency of CD18 may suffer increased infections with early mortality (75).

HSCT has been successfully applied to patients with LAD for more than three decades (77). Similar to other PIDs, results are best when a matched sibling donor is available, but are improving using unrelated or haplo-identical donors.

A trial of gene therapy for LAD-1 was performed in 1992, using a gamma-retroviral vector and no preparative conditioning in two affected children. No significant engraftment of gene corrected cells occurred and no clinical benefit was observed (78). Subsequent studies in a canine model of LAD-1 indicated that relatively low levels of engraftment of healthy matched littermate bone marrow or gene-corrected autologous HSC following non-myeloablative conditioning could prevent subsequent infection (79).

Recently a trial of gene therapy for LAD-1 using a lentiviral vector carrying a normal *ITGB2* cDNA has been opened, sponsored by Rocket Pharmaceuticals. An interim report indicated that the first three LAD-I patients treated had >10% CD18+ neutrophils, with the first patient more than 1 year out from treatment and infection-free, off of prophylactic antibiotics (36).

## Discussion

### Expanding the Applications of Gene Therapy to Additional PIDs

Gene therapies are actively being developed for additional PIDs. Autologous HSC gene therapy using retroviral and lentiviral vectors has been shown to improve disease manifestations in murine BMT models of many PID, including autosomal recessive CGD, hemophagocytic lymphohistiocytosis (HLH), X-Linked Lymphoproliferative (XLP), Immune Dysregulation, Polyendocrinopathy, Enteropathy, X-Linked Syndrome (IPEX), X-linked agammaglobulinemia (XLA) (80). In theory, each of these gene therapies may be translated to the clinic for novel treatments.

Of the more than 20 genes capable of causing human SCID when defective, gene therapy is currently in the clinic for three: ADA SCID, XSCID, and Artemis SCID. A clinical program for gene therapy for Rag1 SCID is underway by collaborative effort in the European Union, using a lentiviral vector with a constitutive retroviral long-terminal repeat enhancer/promoter to drive expression. Their early studies showed high level RAG1 expression is necessary to restore T cell production at sufficiently high levels to prevent autoimmune complications (81). While function of a constitutive, ubiquitous expressed *RAG1* gene may be tempered by the need for simultaneous co-expression of endogenous *RAG2*, it is unknown if ectopic expression of the RAG1 recombinase may lead to toxicities.

IL-7 receptor deficiency SCID is also be a logical candidate for gene therapy. However, mutations in the *IL7R* gene may be drivers in pediatric T ALL (82). The relatively high error rate of lentiviral reverse transcriptase (RT) could result in dominant active mutations being introduced into patient stem cells by RT error during provirus generation. Other genetic forms of SCID (CD3 sub-units, CD45, non-homologous end joining DNA repair factors), could also be treated by gene therapy, but the current complex development process for a clinical product may inhibit derivation of treatment for such ultra-rare disorders.

Several genes involved in PIDs require precise regulation of expression, in developmental or physiologic specific patterns, which is difficult to recapitulate within the size constraints of vector genomes. These include *BTK* for XLA, which is expressed at precise levels at different stages of B cell development and activation and unregulated BTK expression could be oncogenic (83); *CD40L* for X-linked Hyper-IgM syndrome, which has been shown to be oncogenic if constitutively expression (84, 85); *FOXP3* which needs to be expressed in regulatory T cells and their precursors, as well as during T cell activation; and *RAG1*, as discussed above. Gene editing to correct the endogenous genes for each of these disorders could retain the physiologic expression of these genes and is being actively pursued in numerous laboratories.

### Improving Gene Delivery and Expression

Method to produce clinical scale batches of lentiviral vectors for treating large numbers of patients are advancing. The use of suspension cultures of cells in bioreactors is replacing growth of adherent vector producer cells (86). Stable packaging cell lines are being established that would avoid the need for new transfection of vector and packaging plasmids to produce each batch (87). Making stable lentiviral vector packaging cell lines has been challenging because the VSV-G envelope used to pseudotype the vectors and even of some of the HIV genes used to compose the virion may be toxic to packaging cells if constitutively expressed. Inducible gene expression techniques are being used to suppress expression of the vector components until their induction at the time of vector production.

While the basic backbone elements of the lentiviral vectors in current use are essentially those described in the late 1990s (88), the transcriptional regulatory elements used may become more sophisticated. For example, a vector was recently described for the treatment of the PID Immunodysregulation, Polyendocrinopathy, Enteropathy X-Linked (IPEX) that restricts expression of FOXP3 to regulatory T cells after transducing and transplanting HSC by controlling the FOXP3 gene with transcriptional regulatory elements from the endogenous gene (89). Other vectors with precise expression patterns may be developed for other disorders using this approach, which will compete with gene editing strategies that place an exogenous copy of the PID-related gene under control of the endogenous elements by site-specific insertion (90).

### Optimizing Patient Outcomes

For all PIDs, early diagnosis may allow implementation of prophylactic antibiotics and other therapies prior to acquisition of severe infections. The institution of newborn screening for SCID identifies patients in the first weeks of life, which is expected to improve outcomes by allowing early treatment prior to development of infections (91). The development of robust methods for identifying PID-related pathogenic variants, such as whole exome sequencing, are becoming a standard diagnostic tool in medicine and should also contribute to earlier definitive diagnosis and implementation of directed therapies.

Other elements of the gene therapy *per se* are important to improving patient outcomes. Efficacy is increased by giving higher doses of gene-modified HSC, and the use of mobilized peripheral blood stem cells instead of bone marrow improves the starting cell numbers, although rigorous direct comparisons of the outcomes with the two starting cell sources have not been reported. Improved lentiviral vector transduction conditions (higher titer vector, use of transduction enhancer compounds, use of serum-free media that support *ex vivo* HSC survival) can all lead to higher levels of engraftment of gene-corrected HSC. The lessons learned from the use of retroviral vectors with strong enhancer elements that led to insertional oncogenesis and leukoproliferative complications has led to the development and application of safer SIN vectors that lack strong enhancer elements and have significantly reduced risks for causing transformations.

A major limitation of current approaches to both allogeneic HSCT and autologous gene therapy for PIDs is the use of cytoreductive chemotherapy agents to facilitate engraftment of the corrective HSC. These drugs have multiple acute and long-term complications, such as acute organ toxicity (especially hepatic, pulmonary, gastrointestinal) and potential late adverse effects on growth, skeletal and dental development, neurodevelopment, and risks for secondary malignancies. Therefore, there is currently a great deal of effort to develop monoclonal antibody-mediated methods to achieve safe and effective cytoreduction, without the complications of chemotherapy. Antibodies to ckit, CD45, and other stem cell markers are in clinical trials for conditioning, either as unmodified antibody or as antibody drug conjugates with toxins that may increase the elimination of targeted HSC (92, 93). It is hoped that this development will be successful to provide safer methods to facilitate engraftment of HSC to treat PIDs.

### Transition of Gene Therapy to Licensed Medicines

As the field of gene therapy matures, the treatments are moving from early-phase research studies performed at one or a few academic medical centers to become licensed products that are produced commercially and may be administered at multiple institutions with experience in HSCT for PIDs. Ideally, this move to market gene therapies will make them widely available and not require patients and their families to relocate to the few sites that provide them under research trials.

A major issue for commercially prepared gene therapies will be the costs for these treatments. Producing these complex gene and cell therapy products under the strict requirements mandated by FDA, EMA and other oversight entities comes with high costs, in terms of the multiple trained personnel, highly controlled facilities, aspects of a quality assurance program and regulatory requirements. Ultimately, gene therapy will compete with allogeneic HSCT as treatments for PIDs with efficacy, safety and cost all being determining factors of which approach gains acceptance.

### Problems Common to Allogeneic HSCT and Autologous Gene Therapy

While both allogeneic HSCT and autologous gene therapy can alleviate all or most of the immunological and hematological complications of diseases, they often do not address non-hematological complications. For some disorders there may be cross correction of non-hematological tissues by protein made from hematological cells. In ADA SCID, expression of ADA enzyme in a fraction of cells can lower total body pools of adenine nucleotides and rescue survival of ADA-deficient lymphocyte. However, in most cases the non-hematological manifestations, such as the skin or other organs involved in Nemo Syndrome, may not be improved by successful engraftment of HSC with the normal gene. Correction of the gene defect in HSC of Art SCID patients does not correct their general somatic DNA repair defect, and conditioning carries risks for damage to other tissues.

### Summary

In all, gene therapy for PIDs has progressed over the recent decades to be equal to and even better in some cases than allogeneic HSCT. Further improvements in methods should lead to more consistent and reliable efficacy from gene therapy.

## Author Contributions

LK and DK co-wrote the article. All authors contributed to the article and approved the submitted version.

## Conflict of Interest

LK is the recipient of an A. P. Giannini Foundation Postdoctoral Research Fellowship. DK is a member of the Scientific Advisory Board for Orchard Therapeutics that markets Strimvelis and has licensed a lentiviral vector for gene therapy of ADA SCID from the University of California, Los Angeles for which he is an inventor. DK is also the principal investigator for clinical trials discussed in this article on gene therapy for XCGD (California Institute for Regenerative Medicine CLIN2-08231 and Orchard Therapeutics) and LAD-I (California Institute for Regenerative Medicine CLIN2-11480 and Rocket Pharmaceuticals). He is the Chair of the Data Safety Monitoring Board for Leadiant Biosciences for Revcovi PEG-ADA ERT.

## Footnotes

^1^https://ir.orchard-tx.com/index.php/news-releases/news-release-details/orchard-statement-strimvelisr-gammaretroviral-vector-based-gene
